# Navigating the Health Professional Migration Tsunami in the Era of COVID-19 and Globalization: A Call to Action for a Collective and Coordinated Response from Government and Non-Government Organizations

**DOI:** 10.3390/healthcare11070931

**Published:** 2023-03-23

**Authors:** Alessandro Stievano, Rosario Caruso, Franklin Shaffer

**Affiliations:** 1 Centre of Excellence for Nursing Scholarship, OPI Rome, 00136 Rome, Italy; 2 Department of Clinical and Experimental Medicine, University of Messina, 98100 Messina, Italy; 3 Health Professions Research and Development Unit, IRCCS Policlinico San Donato, 20097 Milan, Italy; 4 Department of Biomedical Sciences for Health, University of Milan, 20133 Milan, Italy; 5 CGFNS International, Philadelphia, PA 19104, USA

Migration has always been a part of human history. It has profoundly impacted societies, communities, and cultures [1]. The movement of people from one country to another has brought about changes in demographics, geopolitics discourses, economics, and cultural exchanges [1]. The current global health crisis, provoked by COVID-19, has globally caused a so-called ‘migration tsunami’ in the healthcare workforce. The COVID-19 pandemic has put immense strain on healthcare systems, leading to an even more acute shortage of healthcare professionals in certain countries [2]. This scarcity of professionals has prompted aggressive recruitment tactics by high-income countries, raising ethical concerns about the exploitation of health workers and human trafficking [3]. The pandemic has also highlighted the need for a more resilient and sustainable healthcare workforce, particularly in low- and middle-income countries that are the leading nations that suffer from this problematic situation [4].

This migration tsunami in the healthcare workforce presents a significant challenge to the global community, requiring solutions that balance the need for a robust healthcare workforce with the ethical concerns surrounding the recruitment and deployment of health workers [5]. This editorial aims to discuss the impacts of COVID-19 on healthcare professional flows and provide an analysis of the tests faced by healthcare professionals in the global scenario and the impact of COVID-19 on these flows. This thought-provoking topic is pivotal as the world faces an even greater mobility of healthcare professionals, presenting significant challenges for healthcare systems. Understanding these challenges is crucial in the new normal to ensure and sustain a more resilient and sustainable healthcare workforce.

The COVID-19 pandemic has further exacerbated the shortage of healthcare professionals, leading to increased unsteady flows of health professionals [2]. Consequently, in certain countries, this scarcity has led to aggressive recruitment tactics, including headhunting firms, signing bonuses, and other incentives [5]. Unfortunately, these recruitment tactics are mainly developed with a short-term perspective and raise serious ethical concerns, including exploitation, labor mistreatment, and the unequal distribution of healthcare professionals [6]. However, this short-term approach to the healthcare workforce shortage has long-term consequences, including the depletion of healthcare workers in their home countries and creating complex ethical dilemmas. Consequently, the flow of health professionals must be carefully managed to ensure that healthcare workers are not exploited and that patients receive the best quality of care they need.

Furthermore, the effects of the COVID-19 pandemic on the migration movements of the healthcare workforce must be understood concerning the globalization that has characterized recent decades [7]. While globalization has many positive effects on the healthcare industry, such as improving access to medical technology and expertise, it has also created new challenges for healthcare professionals. In fact, the growth of international trade and the interconnectedness of economies have facilitated the movement of people, goods, and services across borders and this increased mobility has also led to a more remarkable migration of healthcare professionals as countries seek to fill shortages in their healthcare workforce [8]. The increasing competition for healthcare professionals in the global market has resulted in the exploitation of workers, a brain drain from low- and middle-income countries, and a decline in healthcare quality in some communities [9]. The effects of globalization on the migration of healthcare professionals are complex and multifaceted and must be carefully examined to ensure that healthcare systems and professionals are protected.

In our future scenarios, health professionals must deeply recognize how the economics of migration has a profound impact on healthcare systems [5,7]. As countries compete for a limited pool of healthcare professionals, wages and working conditions can become increasingly precarious. This can result in exploitation and a decline in healthcare quality for communities. At the same time, healthcare professionals may be forced to leave their home countries in the search of better economic opportunities, leading to a brain drain and a depletion of the healthcare workforce in already suffering areas. In this framework, regulatory bodies are crucial in controlling health professional flows, ensuring that healthcare systems and professionals are protected [9]. Regulatory bodies should ensure that healthcare professionals are competent, ethical, and regulated through credentialing, codes of ethics, and mutual recognition agreements. These regulations also ensure that patients receive safe and effective care, regardless of where they live or receive treatment.

Considering the current challenging situation, the strategies for navigating the health professional migration tsunami must involve both government and non-government organizations. Both have a crucial role in finding solutions to the challenges posed by the migration of healthcare professionals [5]. An effective collaboration between governments, non-government organizations, and healthcare professional associations is necessary to find solutions and guarantee that all patients receive quality and safe care. In this regard, the United Nations, the World Health Organization, the International Organization for Migration, the Organisation for Economic Co-operation and Development, the International Council of Nurses, and other civil society entities actively seek solutions and best practices for the acculturation and integration of healthcare professionals. One key aspect of these efforts is the promotion of ethical recruitment practices and regulations, which are essential to prevent labor exploitation. The mutual recognition and credentialing of healthcare professionals are also necessary steps in ensuring that healthcare workers are qualified and able to provide quality care.

There is an urgent need for in-depth and multifarious international debate regarding the current migration tsunami in the healthcare workforce and the challenges it poses, including the aggressive recruitment strategies of countries facing a shortage of health professionals and the ethical concerns that arise from these campaigns. Further research and regulatory policies are needed to better regulate the challenges posed by the migration tsunami and find effective solutions. The challenges modeled by the migration of healthcare professionals require a collective, global, and coordinated response. We call on these organizations to work together to address the migration tsunami and find solutions that foster ethical recruitment practices, mutual recognition, and credentialing. Only by working together can we ensure that all patients receive the quality care they need, regardless of where they live. The topic of health professional flows is of vital importance to ensure equity, safety, and adequate healthcare in every country by acknowledging that each nation is interconnected with many others, and ignoring what happens to the more vulnerable contexts can lead to a very negative response loop also involving the wealthiest states. 
